# Titanium Nanotube Modified With Silver Cross-Linked Basic Fibroblast Growth Factor Improves Osteoblastic Activities of Dental Pulp Stem Cells and Antibacterial Effect

**DOI:** 10.3389/fcell.2021.654654

**Published:** 2021-04-01

**Authors:** Abdullkhaleg Ali Albashari, Yan He, Mohammed A. Albaadani, Yangfan Xiang, Jihea Ali, Fengting Hu, Yuan Zhang, Keke Zhang, Lihua Luo, Jianming Wang, Qingsong Ye

**Affiliations:** ^1^School and Hospital of Stomatology, Wenzhou Medical University, Wenzhou, China; ^2^Laboratory for Regenerative Medicine, Tianyou Hospital, Wuhan University of Science and Technology, Wuhan, China; ^3^College of Life and Environmental Science, Wenzhou University, Wenzhou, China; ^4^Center of Regenerative Medicine, Renmin Hospital of Wuhan University, Wuhan, China

**Keywords:** titanium nanotube, dental pulp stem cells, bFGF, implant, silver nanoparticles

## Abstract

Titanium modifications with different silver loading methods demonstrate excellent antibacterial properties. Yet pure silver nanoparticles with limited bioactive properties may delay regeneration of bone surrounding the dental implant. Therefore, loading silver with bioactive drugs on titanium surfaces seems to be a very promising strategy. Herein, we designed a silver (Ag) step-by-step cross-linking with the basic fibroblast growth factor (bFGF) by polydopamine (PDA) and heparin on titanium nanotube (TNT) as its cargo (TNT/PDA/Ag/bFGF) to improve the implant surface. Our results showed that TNT/PDA/Ag/bFGF significantly enhanced the osteogenic differentiation of dental pulp stem cells (DPSCs). It also showed an excellent effect in bacterial inhibition and a reduction of pro-inflammatory factors through inhibition of M1 macrophage activity. These results showed that bFGF cross-linked silver coating on TNTs presented good osteogenic differentiation and early anti-infiammatory and antibacterial properties. Together, this novel design on titanium provides a promising therapeutic for dental implants.

## Introduction

Despite that dental implants have been broadly used in clinics to replace missing teeth and restore oral functions, they have a poor success rate for application with low bone formation surrounding the implant with different surface modifications (Duyck and Vandamme, 2017; Yuan et al., 2019). To encourage the osteoblastic activities and implant osseointegration, numerous studies and attempts have focused on the alterations of surface features and morphology of implant materials via physical and chemical modifications (Marenzi et al., 2019; Siddiqui et al., 2019). Dental pulp stem cells (DPSCs), a type of mesenchymal stem cells (MSCs), have been recognized as a good stem cell source in bone formation. Evidence indicates that initial stem cell adhesion to titanium surfaces is of essential importance in cell proliferation, differentiation, osteogenesis, and osseointegration surrounding the implant (Bandyopadhyay et al., 2019b). Proper surface modification of titanium (Ti) could facilitate the cell-to-Ti interaction and the early osseointegration of implants (Bandyopadhyay et al., 2019a). Also, studies have shown that many growth factors could support cell adhesion and osseointegration with the aid of chemical compounds like heparin, dextran, and hyaluronic acid (Subbiah and Guldberg, 2019). Also, growth factors can maintain and support the renewal and multipotency properties of stem cells (Choi et al., 2018). Bone morphogenetic protein 2 (BMP-2) is one of the most studied growth factors in bone formation that are used with Ti (Haimov et al., 2017). Except for the obvious osteogenic capacity of BMP-2, it does not have a strong supportive role in the proliferation and differentiation of stem cells as the basic fibroblast growth factor (bFGF) does (Kang et al., 2019). It has been proved that bFGF is one of the influential mitogens for many MSCs (Luo et al., 2020).

On the other hand, due to the naturally occurring oral microorganisms, it is important and challenging in clinical application to control the infection and promote osseointegration (Krastl and Amato, 2019). Many antibacterial drugs have been incorporated on titanium surfaces to prevent infections, such as amoxicillin, doxycycline, and cephalexin (Orapiriyakul et al., 2018). Limited by the potency of induced drug resistance, bioactivity, and loading capacity, the choice of drugs requires a sustained release of bioactive antibiotics (14–16). As a previous work indicated (Chouirfa et al., 2019), properly designed surface modification on Ti would be a possible solution to control-release the antibacterial agents, prohibit bacterial proliferation, and prevent biofilm formation. Because different nanoparticles are favorable bactericidal agents owing to their antibacterial wide-spectrum activity, good stability, low cytotoxicity, and high efficiency, Ag nanoparticles can penetrate biofilms and provide antimicrobial effects on both single-species biofilm of gram-positive and gram-negative bacteria and multi-species of oral biofilms (He et al., 2013, 2014; Peterson et al., 2015).

Current loading methods, including physical binding loading, dip loading, sputter loading, electrophoresis loading, and chemically conjugated loading, have several shortcomings like shape dependence, difficulty fabricating process, environmental pollution, expensive instruments, and harsh processing conditions. To get the better stability of loading multifactors on titanium, covalent cross-linking grafting has brought about step-by-step cross-linking, early cross-linking, and post-cross-linking. Unfortunately, this type of silver and bFGF step-by-step cross-linking delivery system on titanium nanotube (TNT) has not been developed. In this work, we fabricated TNTs coated with polydopamine (PDA) and Ag-bFGF cross-linking heparin binding. It is speculated that an Ag-bFGF cross-linking binding on titanium will control infection and sustainably promote bone formation. We believe that this strategy may inspire the design of step-by-step cross-linking loading for dental implants on titanium in the future.

## Materials and Methods

### Materials and Chemicals

Titanium sheets (3 mm thick) were obtained from the Northwest Institute for Nonferrous Metal Research (Xi’an, China). Silver nitrate, ammonium fluoride, glycerol, heparin, paraformaldehyde (PFA), 2-(*N*-morpholino)ethanesulfonic acid, *n*-hydroxysuccinimide, 1-ethyl-3-(3-dimethylaminopropyl)carbodiimide, and toluidine blue were obtained from Aladdin Industrial Corporation (Shanghai, China). Dimethyl sulfoxide, Lysogeny broth (LB) medium, Triton X-100, 3-(4,5-dimethylthiazol-2-yl)-2,5-diphenyltetrazolium bromide (MTT), and Tris–HCl were obtained from Solarbio (China). Interleukin-6 (IL-6) detection IL-6 antibody was purchased from Affbiotech (DF6087, United States). Bicinchoninic acid kit (BCA kit), nitric oxide kit, 4′,6-diamidino-2-phenylindole (DAPI), and *p*-nitrophenyl phosphate assay kit were bought from Beyotime Institute of Biotechnology (Shanghai, China). Cell Counting Kit-8 (CCK-8) was purchased from Dojindo (Kumamoto, Japan). Recombinant bFGF, minimum essential medium α (MEM-α), Dulbecco modified Eagle’s minimal essential medium (DMEM), phosphate-buffered saline (PBS), fetal bovine serum (FBS), and penicillin–streptomycin were bought from Gibco (Invitrogen, United States). Osteogenic medium was purchased from Cyagen, United States. ELISA kit for bFGF was purchased from Westang System (Shanghai, China). Sangon kit for RNA extraction was purchased from Sangon Biotech (Shanghai, China). Reverse RNA kit and PrimeScript RT kits were purchased from Takara Bio Inc (Kyoto, Japan).

### Sample Preparation

**FIGURE 1:**
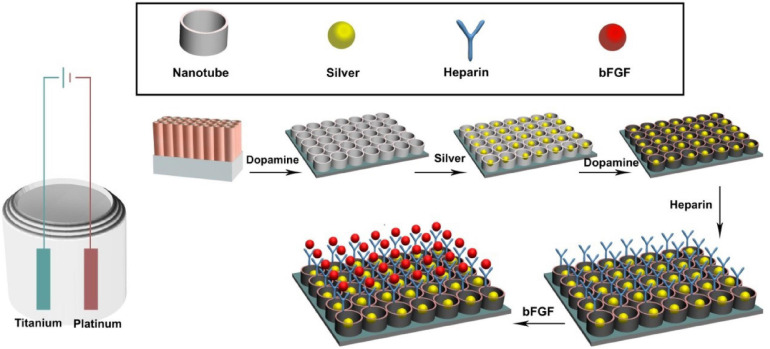
Experimental design of the *in vitro* experiments. The titanium sheet was cut into the size of 1 cm × 1 cm pieces. Then the titanium surface was modified to titanium nanotube, coated with dopamine, and cross-linked with heparin and basic fibroblast growth factor (bFGF).

As shown in Figure 1, sample surfaces went through a series of modifications to contain active components. Individual surface modifications are explained below.

#### Titanium Nanotube Preparation and Modification

The surface of the titanium (Ti) sheet was polished, smoothed, and then cleaned with acetone, ethanol, and distilled water in 300-W ultrasound for 15 min. Platinum foil and Ti sheet were placed as the cathode and anodic for oxidation modification, respectively. The distance between two electrodes was set at about 3 cm. TNT modification was performed in a glycerol electrolyte containing 0.51% wt ammonium fluoride and 8.1% wt water. First, the Ti sheet was oxidized at 20 V for 3 h followed by 2 min oxidation at 170 V. Then the Ti sheet was ultrasonicated (at 90 W, in water bath) for 5 min. Finally, the Ti sheet was oxidized for 20 min at 20 V to form nanotubes on the surface (Shen et al., 2019).

#### Ag Cross-Linking on Ti Surfaces

Silver ion was coated onto TNT surfaces in two methods, direct coating and via a PDA film. *Direct coating*: The TNT sheet was immersed in a silver nitrate solution (1.5 mg/ml) for 20 min in the dark, rinsed five times with deionized water, and air-dried. Then ultraviolet light was applied with a high-voltage Hg lamp for 20 min per side to receive TNT/Ag samples. *Coating via PDA film*: The TNT sheet was immersed in 2 mg/ml of dopamine in 10 mM of Tris–HCl buffer (pH 8.5) for 24 h at room temperature, washed by deionized water five times, and air-dried. Then this TNT/PDA sheet was coated with silver nitrate using the direct coating method to receive TNT/PDA/Ag samples.

#### Basic Fibroblast Growth Factor Functionalized Ti Samples via Heparin

To functionalize sample surfaces with bFGF, samples were immersed in 1 mg/ml of dopamine in 10 mM of Tris–HCl solution (pH = 8.5) for 12 h at room temperature in the dark, washed five times with deionized water, and air-dried. To aminate sample surface with heparin (HP), samples were immersed in a heparin solution [1 mg/ml heparin in 0.1 M of 2-(*N*-morpholino)ethanesulfonic acid solution (pH 5.6), 0.18 mg of *n*-hydroxysuccinimide, and 0.59 mg of 1-ethyl-3-(3-dimethylaminopropyl) carbodiimide] for 24 h followed by deionized water rinse and then air-dried. To functionalize samples with bFGF, 120 ng of bFGF was dissolved in 0.1 M of ethanesulfonic acid solution (pH 5.6), pipetted on the surface and left for 24 h at room temperature in the dark, washed by deionized water, and air-dried. To study the release of bFGF on heparinized sample (TNT/PDA/HP), bFGF-coated TNT and TNT/PDA/Ag samples were used as control.

### Characterization of Ti Samples

The morphologic characterization of sample surfaces, coated with platinum, was examined by scanning electron microscopy (SEM; Hitachi, S2300, Japan) at the Wenzhou Institute of Biomaterials and Engineering, where the diameter of nanotubes was measured and compared. The composition of samples was assessed by the energy-dispersive X-ray spectroscopy (EDS). To evaluate the surface hydrophobicity, we performed contact angle studies on sample surfaces using deionized water.

### Loading of Heparin Assay

To determine the quantity of coated heparin on sample surfaces, a toluidine blue protocol was adopted (Kim et al., 2011). Briefly, samples were immersed in 1 ml of PBS (pH 7.4) containing 0.005% toluidine blue solution for 40 min under gentle shaking at room temperature. Then the absorbance value of the solution was read using a spectrophotometric microplate reader at 620 nm (Varioskan LUX, Thermo Fisher Scientific, United States).

### Protein Adsorption Assay

To evaluate protein adsorption on the substrates, bovine serum albumin (BSA) was used as a representative protein instead of bFGF (Kim et al., 2011). Five hundred microliters of 1 mg/ml BSA in PBS was added onto each sample for 2 h. Then, samples were washed once with PBS. BCA kit assay was performed, and the absorbance was measured at 570 nm using a microplate reader.

### Release of Basic Fibroblast Growth Factor

To study the accumulative release of bFGF from assorted substrates (TNT, TNT/PDA/Ag, and TNT/PDA/HP), samples were soaked in 1 ml of PBS (pH 7.4) (100 rpm, 37°C). At established time points (0, 3, 6, 9, 12, 15, 18, and 21 days), the solution was collected and replaced with 1 ml of new PBS. Samples were kept at −20°C for quantitative analysis by bFGF ELISA kit according to the manufacturer’s protocol.

### Cellular Evaluations on Modified Ti Samples

#### Culture and Viability of Dental Pulp Stem Cells on Ti Samples

The isolation, culture, and identification of DPSCs were described in our previous paper (Luo et al., 2018). The use of DPSCs described in this paper was reviewed and approved by the Ethics Committee of the School and Hospital of Stomatology, Wenzhou Medical University (No. WYKQ2018008). Sterile samples (1 cm × 1 cm) were placed in 24-well plates and seeded with DPSCs (1 × 10^4^ cells/cm^2^, passage 5) for 2 h at 37°C and 5% CO_2_ humidified atmosphere. Then 1 ml/well of MEM-α medium supplemented with 10% FBS and 1% penicillin–streptomycin was used to start cell culture. The first passage of DPSCs was characterized by flow cytometry using the antibodies of human CD34, CD45, CD73, and CD105 according to standard protocols. The data were evaluated with CytoFLEX flow cytometers (Beckman Coulter, California, United States). The DPSCs were evaluated by immunofluorescence staining with CD44, CD146, and CD31. After 1 and 3 days, CCK-8 solution was applied to evaluate the viability of cells adhering to sample surfaces.

#### Morphology of Dental Pulp Stem Cells on Modified Ti Samples

To evaluate DPSCs on the surfaces, observation on cell morphology via SEM and immunofluorescence microscopy was performed. After 1-day culture of DPSCs on sample surfaces, cells were fixed with 4% PFA for 1 h, washed three times with PBS, dehydrated in a series of ethanol from 30 to 100%, and air-dried at 37°C. The samples were platinum coated and examined by SEM.

To visualize DPSCs on sample surfaces after 1- and 3-day culture, samples were washed with PBS three times, fixed with 4% PFA for 15 min, and rinsed with PBS three times. Cells were permeabilized by 0.1% Triton X-100 in PBS for 5 min and 5% BSA for 30 min at 37°C. Then DPSCs were labeled by 1 mg/ml of phalloidin-TRITC and 2 mg/ml DAPI. Five random locations per sample were photographed by a fluorescence microscope (Eclipse 80i, Nikon, Japan).

#### Osteogenic Capacity of Dental Pulp Stem Cells on Modified Ti Samples

##### Alkaline phosphatase activity

To evaluate the osteogenic differentiation of DPSCs on the sample surfaces, cells grown on sample surfaces were cultured with the osteogenic medium. On days 7 and 14, the alkaline phosphatase (ALP) activity of the cells was assessed. Briefly, cells were lysed by sonication within 1 ml of DNA-free H_2_O containing 0.02% wt Triton X-100 for 45 min at room temperature. Total protein concentration and ALP activity of DPSCs were quantified with BCA kit (570 nm) and *p*-nitrophenyl phosphate assay kit (490 nm) by the microplate reader.

##### Mineralization level

Dental pulp stem cells grown on sample surfaces were cultured with osteogenic medium for 21 days. Then the samples were fixed with 4% PFA. To evaluate the formation of calcified nodules, samples were stained with Alizarin red S (pH 4.1), treated with 10% v/v acetic acid and 10% v/v ammonium hydroxide, and measured at 405 nm with the microplate reader. The cells were fixed for 30 min with 2.5% glutaraldehyde and dehydrated by gradient methanol (30, 40, 50, 70, 80, 90, 95, and 100%). The cells were observed using SEM.

##### Expression of osteogenic genes

Dental pulp stem cells were cultured on sample surfaces for 10 days using osteogenic medium. Total RNA was extracted using Sangon kit. RNA extractions were performed according to the manufacturer’s protocol. Then 2 μg of extracted RNA was reversely transcribed to cDNA using cDNA Takara Reverse kits. Gene expression of ALP, runt-related transcription factor 2 (RUNX), osteocalcin (OCN), and osteopontin (OPN) were measured with corresponding primers (Table 1). Expression levels of all genes were normalized to GAPDH.

**TABLE 1:** Expression of osteogenic genes.

ALP-F	GCTATCCTGGCTCCGTGCT
ALP-R	ACAGATTTCCCAGCGTCCTT
OCN-F	GCAAAGGTGCAGCCTTTGTG
OCN-R	GGCTCCCAGCCATTGATACAG
Runx2-F	GCGGTGCAAACTTTCTCCAG
Runx2-R	TGCTTGCAGCCTTAAATGACTC
OPN-F	TTTTGCCTCCTAGGCATCACC
OPN-R	TGGAAGGGTCTGTGGGGCTA
GAPDH-F	ATGGGCAGCCGTTAGGAAAG
GAPDH-R	GATCTCGCTCCTGGAAGATGG

### Antibacterial Property of Modified Ti Samples

#### Bacterial Culture and Short-Term Antibacterial Assay of Ti Samples

*Escherichia coli* ATCC25922 (*E. coli*) and *Staphylococcus aureus* ATCC35984 (*S. aureus*) were separately seeded on modified Ti surfaces at a density of 1 × 10^6^/cm^2^ and cultured with LB medium for 12 h at 37°C aerobically. Then, samples were fixed with 2.5% glutaraldehyde, dehydrated by gradient methanol, coated with platinum, and visualized by SEM. To qualitatively evaluate the antibacterial property of these Ti samples, disk diffusion assay was performed to compare the inhibition zone and bacterial colony free zone. One milliliter of 10^6^ cells/ml bacterial solution per plate was inoculated on LB agar plate. Modified Ti samples were placed on the agar plates and incubated aerobically for 24 h at 37°C. The inhibition zone was photographed and compared (Cai et al., 2018).

#### Antibacterial Activity of Pre-released Ti Samples

To pre-release coated active components on modified Ti sheets, all samples were drenched in PBS (pH 7.4) for 1, 3, and 7 days. Then these pre-released Ti samples were placed in a 24-well plate and inoculated with *E. coli* or *S. aureus* (1 × 10^6^/cm^2^) using LB medium in an aerobic incubator at 37°C. After 24 h, the medium was replaced by a mixture of 50 μl of MTT (5 mg/ml) and 450 μl of fresh medium per well to evaluate the viability of the bacteria. After 2-h incubation at 37°C in the dark, the mixture was replaced with 500 μl of dimethyl sulfoxide and measured at 540 nm by the microplate reader.

#### Titanium Modification on *Porphyromonas gingivalis* Associated With Peri-Implantitis

*Porphyromonas gingivalis* (ATCC 33277) was cultured in brain heart infusion (BHI) broth containing 0.001% hemin, 0.0001% vitamin K, and 5 mg/ml of yeast extraction in an anaerobic chamber (85% N_2_, 10% H_2_, and 5% CO_2_ at 37°C; GeneScience Anaerobox IV, United States). For SEM and MTT assay, 1 × 10^6^ colony-forming units (CFU) ml^–1^ of *P. gingivalis* was evaluated. All the modified titanium surfaces were cultured in 24-well plates with each well containing 1 ml of BHI, and then *P. gingivalis* is inoculated. The cells were fixed for 30 min with 2.5% glutaraldehyde and dehydrated by gradient methanol (30, 40, 50, 70, 80, 90, 95, and 100%). The cells were observed by using SEM after 24 h. To pre-release coated active components on modified Ti sheets, all samples were drenched in PBS (pH 7.4) for 1, 3, and 7 days. For MTT assay on days 1, 3, and 7, the medium was replaced by a mixture of 50 μl of MTT (5 mg/ml) and 450 μl of fresh medium per well for 2 h in the dark. Then, the mixture was replaced with 500 μl of dimethyl sulfoxide and measured at 540 nm by the microplate reader. All surfaces were placed in an anaerobic chamber (85% N_2_, 10% H_2_, and 5% CO_2_ at 37°C).

### Anti-inflammatory Property of Modified Ti Samples

#### RAW 264.7 Cell Culture and Pro-inflammatory Expression

RAW 264.7 cell was obtained from the Wenzhou Institute of Biomaterials and Engineering (Wenzhou, China) and cultured with DMEM containing 10% FBS and 1% penicillin–streptomycin. RAW 264.7 cells were seeded on Ti sample surfaces at 1 × 10^5^ cells/cm^2^ in 24-well plates and cultured for 24 h. Then the cells were challenged by culture medium supplemented with 500 ng/ml of lipopolysaccharide (LPS). After 12 h, cells were fixed with 4% PFA and washed with PBS thrice followed by 5 min permeabilization with 0.1% Triton X-100 and 30 min with 5% BSA at 37°C. Pro-inflammatory factor, IL-6, was stained by an immunofluorescence label. Nuclei were stained with 2 mg/ml of DAPI. Images were taken by the fluorescence microscope.

#### Expression of Pro-inflammatory Genes

RAW 264.7 cell was challenged the same way as above. Total RNA was extracted from cells. Relative mRNA expression of pro-inflammatory factors, IL-6, and TNF-α was assessed. cDNA was transcribed from RNA by using Prime Script RT kit. Quantitative PCR samples were performed using a total volume of 20 μl with identical primers (Table 2) and PCR SYBR Green Kit (95°C for 30 s, following by 39 cycles of 95°C for 5 s and 60°C for 30 s). Data were normalized with GAPDH, analyzed using the 2^–ΔΔCT^ method, and expressed in the mean value of each group.

**TABLE 2:** Expression of pro-inflammatory genes.

TNF-α-F	CCAGGCAGGTTCTGTCCCTT
TNF-α-R	ATAGGCACCGCCTGGAGTTC
IL-6-F	CTGGAGCCCACCAAGAACGA
IL-6-R	GCCTCCGACTTGTGAAGTGGT
GAPDH-F	GGATGCAGGGATGATGTTC
GAPDH-R	TGCACCACCAACTGCTTAG

#### Inhibition of Nitric Oxide Production

Nitric oxide production has been regarded as an effective indicator to study the inflammatory status of macrophages (Palmieri et al., 2020). To quantify the level of nitric oxide, RAW 264.7 cells were seeded at 1 × 10^5^ cells/cm^2^ in 24-well plates on sample surfaces and cultured overnight with DMEM containing 10% FBS and 1% penicillin–streptomycin. Then medium was replaced with FBS-free DMEM supplemented with 500 ng/ml of LPS for 1 and 3 days. The nitrite was determined in culture media according to protocol instruction kit (s0021, Beyotime, China). The total reaction was 50 μl of medium mixed with 50 μl of Griess Reagent I in 96-well plate and applying 50 μl of Griess Reagent II. Then the absorbance was measured at 540 nm in a microplate reader (Varioskan LUX, Thermo Fisher Scientific, United States).

### Statistical Analysis

One-way analysis of variance (ANOVA) was used to analyze the data; then, we analyzed the homogeneity of variance by Levene test. If the significance of Levene test was >0.05, we used Tukey’s test for multiple comparisons; if not, we checked the rest of the results. And statistical significance was set as *p* < 0.05. Statistical significance was defined as ^∗^*p* < 0.05, ^∗∗^*p* < 0.01, and ^∗∗∗^*p* < 0.001.

## Results and Discussion

### Characterization and Multipotency Analysis of Dental Pulp Stem Cells

**FIGURE 2:**
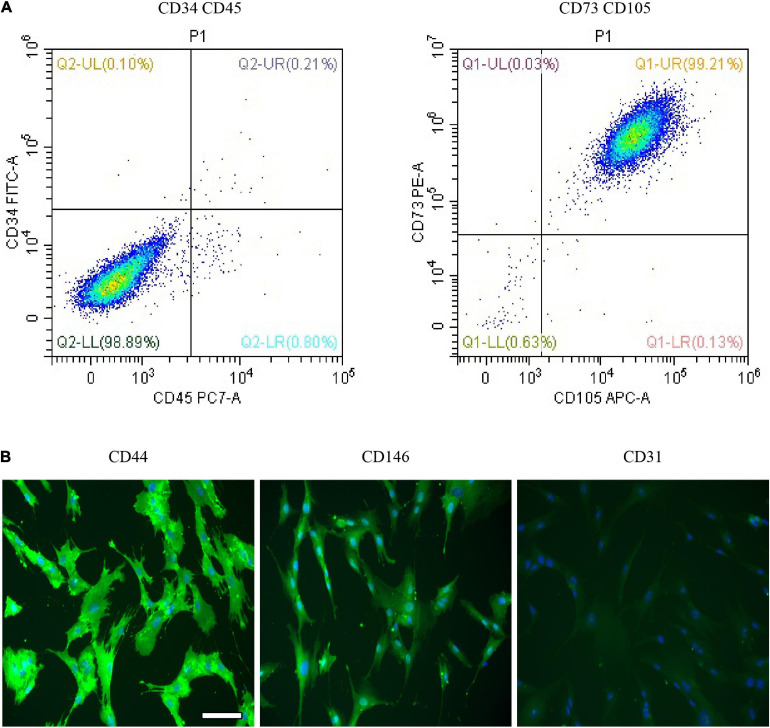
Characterization and multipotency analysis of dental pulp stem cells (DPSCs). **(A)** The expression of surface markers of DPSCs. DPSCs positively expressed phenotypic markers CD105 and CD73 but negatively expressed CD34 and CD45. **(B)** Immunofluorescence expression of DPSC. DPSCs showed a positive expression for CD44 and CD164 but negative expression for CD31. Scale bar for **(B)**, 100 μm.

Dental pulp stem cells are one type of MSCs and possessed the characteristics of MSCs; for example, DPSCs have multidifferentiation potentials and express MSC-like markers. For the DPSC identification, flow cytometry analysis was performed to examine the MSC-like properties of DPSCs. As presented in Figure 2A, the flow cytometry results showed that DPSCs positively expressed CD105 and CD73 phenotypic markers but negatively expressed CD34 and CD45. Also, the result proposed that DPSCs positively expressed the immunofluorescence for CD44 and CD164 but negatively expressed CD31 (Figure 2B). Studies showed that DPSCs have positively expressed MSC-like phenotypic markers such as STRO-1, CD146, CD105, CD90, and CD73 and negatively expressed hematopoietic lineage molecules, including HLA-DR, CD45, CD14, and CD34 (Gronthos et al., 2002; He et al., 2020).

### Characterization of Samples

**FIGURE 3:**
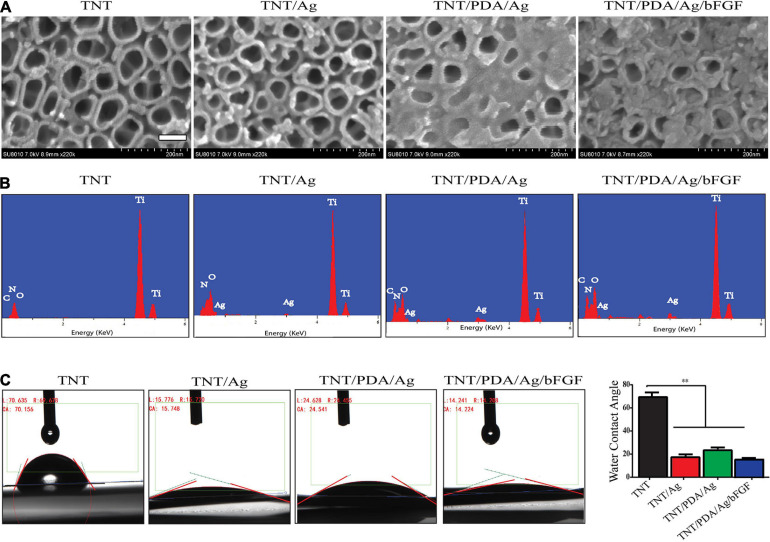
Characterization of modified Ti samples. **(A)** SEM images of Ti challenged at 20 V: TNT, TNT/Ag, TNT/PDA/Ag, and TNT/PDA/Ag/bFGF. Ag nanoparticles on pores could be detected at nanotube inner surfaces in TNT/Ag. TNT/PDA/Ag and TNT/PDA/Ag/bFGF were partially covered with adherent particles, and the number of these particles increased in TNT/PDA/Ag/bFGF. Scale bar represents 500 nm. **(B)** Energy-dispersive X-ray spectroscopy (EDS) of modified Ti samples. TNT was mainly composed of titanium (Ti) with traces of nitrogen (N), oxygen (O), and carbon (C). Ag could be detected in TNT/Ag, TNT/PDA/Ag, and TNT/PDA/Ag/bFGF. **(C)** Water contact angle study indicated a hydrophilic surface feature in TNT/Ag, TNT/PDA/Ag, and TNT/PDA/Ag/bFGF. Data are presented as mean ± standard deviation; ^∗∗^*p* < 0.01.

According to measurement on SEM images, the nanotube diameter of TNT, TNT/Ag, TNT/PDA/Ag, and TNT/PDA/Ag/bFGF was 79.8 ± 10.3, 70.6 ± 7.1, 60.6 ± 8.9, and 58.6 ± 7.4 nm, respectively (Figure 3A). The nanoparticles of Ag on pores could be detected at nanotube inner surfaces in the TNT/Ag group. The wall thickness and pore size difference could be attributed to a different kind of coating structure. Some studies show different surface modifications of the TNT implants because of the excellent response to surface coating, which may encourage effective bonding between cells and implants and short healing of bone surrounding implants (Meyers and Grinstaff, 2012; Mokhtari et al., 2018; Bandyopadhyay et al., 2019b).

To study the formation of TNT/PDA/Ag/bFGF composite, EDS was performed. In the EDS study, different Ti modification samples were focused, and the peaks are shown in Figure 3B. All the Ti, C, O, and N could be seen in the EDS spectrum in all groups. Ag can be seen only in TNT/Ag, TNT/PDA/Ag, and TNT/PDA/Ag/bFGF.

From water contact angle study, there was a decrease of hydrophobicity on modified TNT surfaces (*p* < 0.05, Figure 3C), indicating that a cell adhesion-friendly surface on TNT samples was achieved with modifications introduced in this work. A previous work has shown that cell adhesion, proliferation, differentiation, and biologic activity of protein adhesion improved as the surface hydrophobicity of titanium decreased (Martin et al., 1995; Neoh et al., 2012).

### *In vitro* Loading and Release Tests of Modified Ti Samples

**FIGURE 4:**
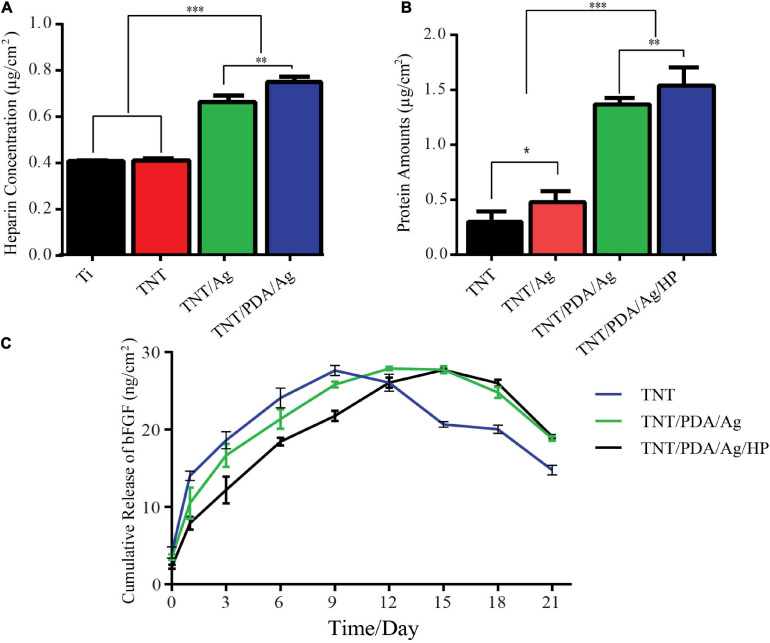
*In vitro* loading and release tests of modified Ti samples. **(A)** Heparin loading assay: samples were immersed in 1 ml of phosphate-buffered saline (PBS) (pH 7.4) containing 0.005% toluidine blue solution for 40 min, and solutions were measured at 620 nm. TNT/PDA/Ag surface showed the highest heparin loading capacity. **(B)** Bovine serum albumin (BSA) release of modified Ti samples: a total of 500 μg of BSA per modified Ti samples was added and left for 2 h. The adsorbed amount of BSA was quantified using a bicinchoninic acid kit (BCA) kit assay (570 nm). TNT/PDA/Ag/HP showed the highest protein adsorption among all modified surfaces. **(C)** Cumulative release of basic fibroblast growth factor (bFGF) from modified Ti samples in PBS (pH: 7.4) at 37°C measured by ELISA. Both TNT/PDA/Ag and TNT/PDA/Ag/HP displayed a slow release, with the latter being even more slowly. TNT, TNT/PDA/Ag, and TNT/PDA/Ag/HP reached the release peak on days 9, 12, and 15, respectively. Data are presented as mean ± standard deviation; ^∗^*p* < 0.05, ^∗∗^*p* < 0.01, and ^∗∗∗^*p* < 0.001.

To profile the heparin loaded onto Ti, TNT, TNT/Ag, and TNT/PDA, toluidine staining was performed. TNT/PDA/Ag surface showed the highest heparin release (Figure 4A), indicating that PDA could significantly facilitate heparin loading. This would greatly impact the release of bFGF in that bFGF was functionalized on modified Ti surfaces via heparin.

Instead of bFGF, BSA was applied to perform the protein adsorption on modified Ti surfaces using a BCA kit. We confirmed that TNT/PDA/HP was more effective in immobilizing BSA (1.55 μg/cm^2^) than TNT (0.32 μg/cm^2^), TNT/Ag (1.41 μg/cm^2^), and TNT/PDA/Ag (0.60 μg/cm^2^) (Figure 4B). Protein adsorption on titanium surface is important because it affects the early cell response to surfaces, including cell proliferation and differentiation (Mata et al., 2003; Grigoriou et al., 2005). Rivera and co-workers proved that cell adhesion and proliferation capacity increased as more protein adsorbed on titanium surfaces (Rivera-Chacon et al., 2013).

At predetermined time intervals, the release kinetics of bFGF immobilized on the TNT/PDA/Ag/HP was analyzed by ELISA kit (Figure 4C). TNT showed the fastest release of bFGF, reached the peak on day 9, and failed to maintain a plateau. On the contrary, TNT/PDA/Ag reached release peak on day 12 and TNT/PDA/HP on day 1. Both TNT/PDA/Ag and TNT/PDA/HP displayed a slow release with the latter being even more slowly. It seemed that heparin coating could further slow down the release of bFGF from Ti surface. Also, heparin has been known to protect growth factor from degradation by decreasing the noggin binding (Ruppert et al., 1996; Luo et al., 2018). It combatively inhibits the noggin binding to osteoblasts, resulting in a prolonged half-life of growth factor. The study reported that heparin could extend the lifetime of BMP-2 by 20 times longer (Zhao et al., 2006). Yang et al. (2015) showed that titanium coated with heparin and BMP-2 promoted the proliferation of MG-63 human osteosarcoma cells and their osteoblast differentiation.

### Viability and Proliferation of Dental Pulp Stem Cells on Modified Ti Samples

**FIGURE 5:**
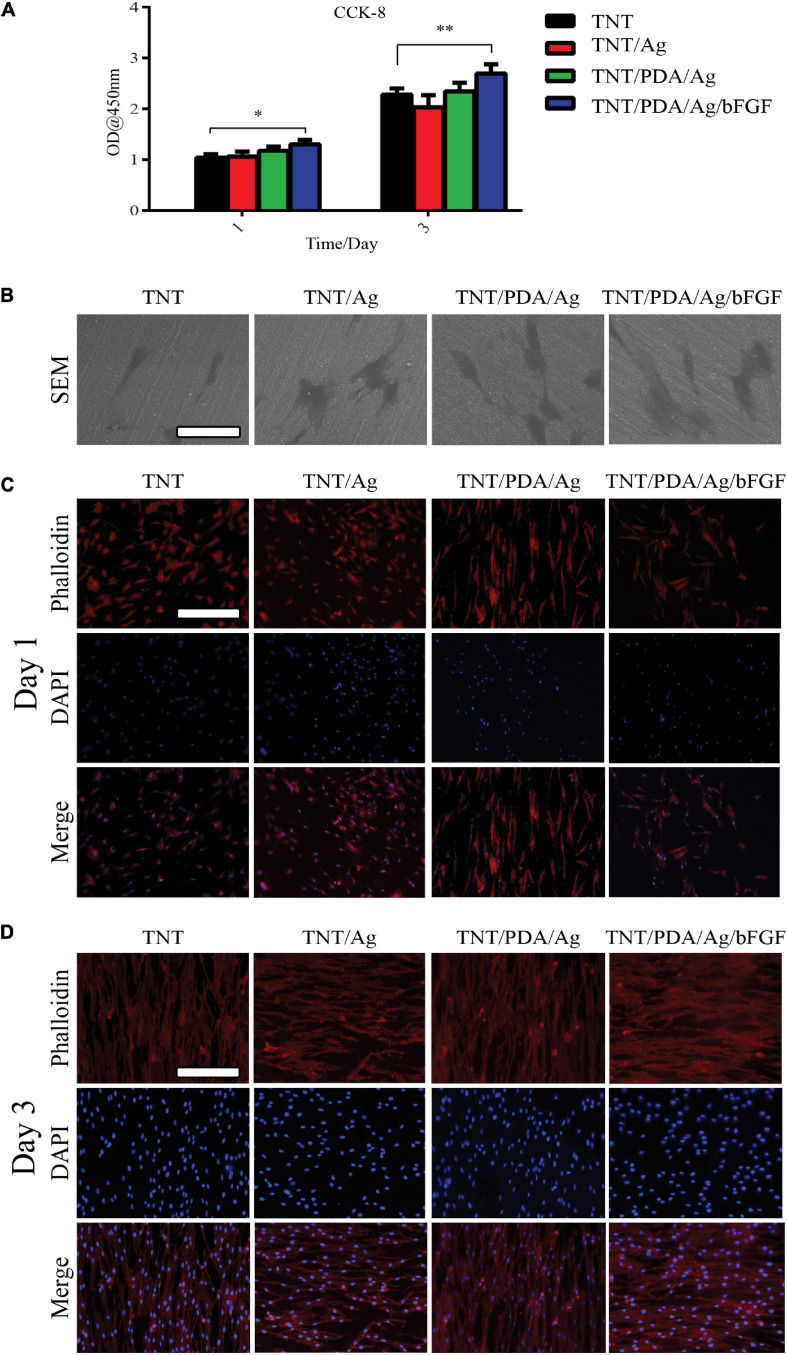
Features of dental pulp stem cells (DPSCs) on modified Ti samples. **(A)** Determined by Cell Counting Kit-8 (CCK-8), on both days 1 and 3, DPSCs showed the highest viability on TNT/PDA/Ag/bFGF. **(B)** After 1-day culture of DPSCs on sample surfaces, cells were processed for SEM, which confirmed that DPSCs spread well on all modified Ti surfaces. Scale bar represents 20 μm. **(C,D)** To visualize DPSCs on sample surfaces after 1- and 3-day culture, cells were labeled by phalloidin-TRITC (red) and DAPI (blue). During the first day of culture of DPSCs on modified Ti surfaces, cells on TNT/PDA/Ag and TNT/PDA/Ag/bFGF were aligned. On day 3, DPSCs were well aligned in high density on all surfaces. Scale bar represents 20 μm. Data are presented as mean ± standard deviation; ^∗^*p* < 0.05, ^∗∗^*p* < 0.01.

The viability of DPSCs that adhered on modified Ti surfaces was examined using a CCK-8 assay after 1- and 3-day culture (Figure 5A). On both days 1 and 3, TNT/PDA/Ag/bFGF showed the highest viable results. Also, the CCK-8 results indicated an increase of cell number after 3 days growth on the surfaces. SEM image confirmed that DPSCs spread well on Ti surfaces after 1-day culture (Figure 5B). Immunofluorescence staining of DPSCs after 1- and 3-day culture visualized the cells on the surfaces (Figures 5C,D). Compared with that on day 1, the cell density was increased on day 3, corresponding to the CCK-8 results. Previous studies indicated that bFGF assisted cell adhesion on the hydrogel (Yan et al., 2010; Guo et al., 2019). On day 1, DPSCs on TNT/PDA/Ag showed alignment on the surface. This observation was also reported in others’ work. PDA-coated TNTs decreased the pore size of the nanotube and supported cell adhesion (Ko et al., 2013; Kao et al., 2015). In our study, we discovered that DPSCs on all Ti surfaces aligned in one direction on day 3.

### Osteogenic Differentiation of Dental Pulp Stem Cells on Modified Ti Samples

**FIGURE 6:**
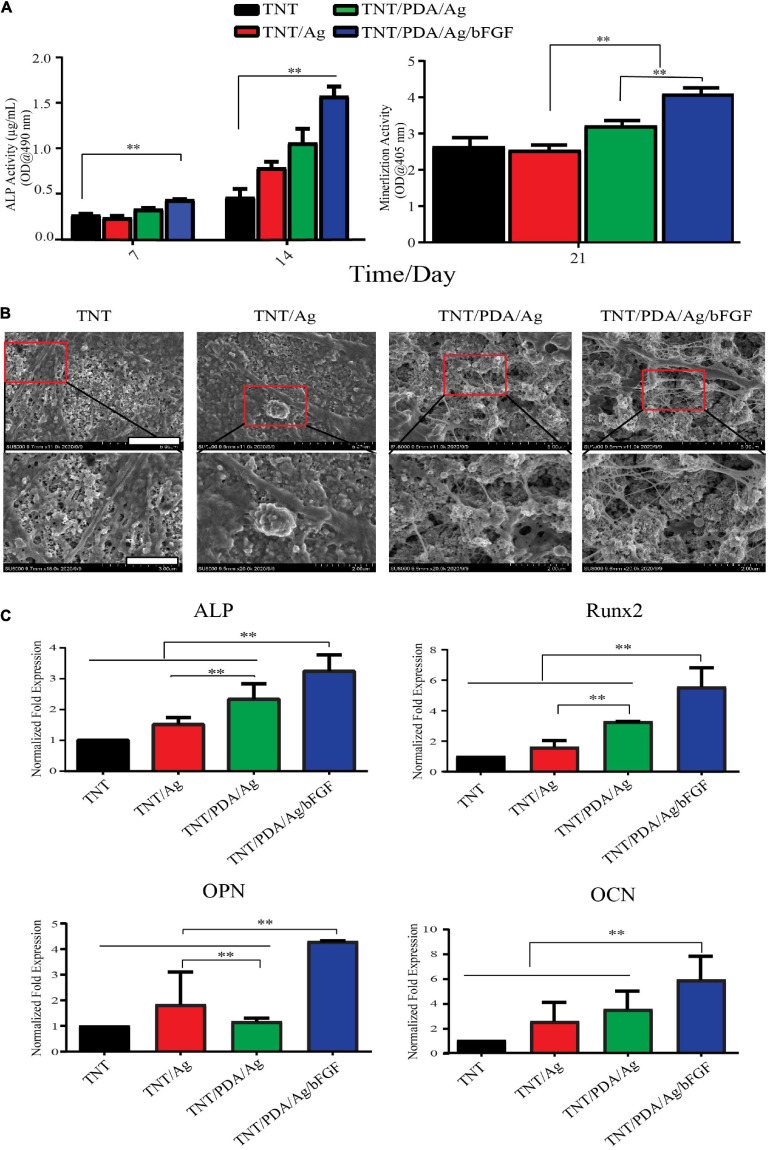
Osteogenic differentiation of DPSCs on modified Ti samples. **(A)** ALP activity of DPSCs cultured on TNT/PDA/Ag/bFGF was significantly higher than that on other surfaces on days 7 and 14. Mineralization assay stained with Alizarin red S indicated that DPSCs grown on TNT/PDA/Ag/bFGF showed the highest calcium deposition among all surfaces. **(B)** After 21-day culture of DPSCs on sample surfaces, cells were prepared and examined by SEM. Calcium deposition on TNT/PDA/Ag/bFGF was significantly higher in all surfaces. Scale bars represent 5 and 2 μm in the upper and lower panels, respectively. **(C)** Relative mRNA expression of osteogenic genes (ALP, OPN, RUNX, and OCN) were normalized to GAPDH and compared among all groups. Osteogenic gene expression of DPSCs cultured on TNT/PDA/Ag/bFGF was the highest. Data are presented as mean ± standard deviation; ^∗∗^*p* < 0.01. DPSCs, dental pulp stem cells; ALP, alkaline phosphatase; OPN, osteopontin; RUNX, runt-related transcription factor 2; OCN, osteocalcin.

The ALP evolution of the DPSCs was investigated after 7 and 14 days on the modified samples. In Figure 6A, significant differences were shown between the ALP of DPSCs cultured on TNT containing silver with bFGF and other groups. The ALP activity of DPSCs co-cultured on the TNT/PDA/Ag/bFGF was higher than that of others cultured on TNT/PDA/Ag during the culture time. DPSCs grown on the TNT/PDA/Ag had significantly higher ALP activity than those grown on TNT/Ag for 7 and 14 days. The calcium amount deposited by DPSCs differentiated on each group was tested at 21 days of cell culture. By using SEM, the results of the amount of calcium deposition significantly show that DPSCs cultured on the TNT/PDA/Ag had higher amounts of calcium than the ones cultured on TNT/Ag (Figure 6B). Clearly, differences were shown in the calcium deposition level between DPSCs cultivated on TNT/PDA/Ag/bFGF and other surfaces (Figure 6B). The results of ALP, OPN, and OCN gene expression by DPSCs were evaluated by using real-time PCR after 14-day culture. In Figure 6C, significant differences were observed between gene expression by DPSCs that differed on the TNT/PDA/Ag and other groups. The ALP, OPN, and OCN by DPSCs cultivated on TNT/PDA/Ag were significantly greater than those cultivated on TNT/Ag.

### Antibacterial Properties of Modified Ti Samples

**FIGURE 7:**
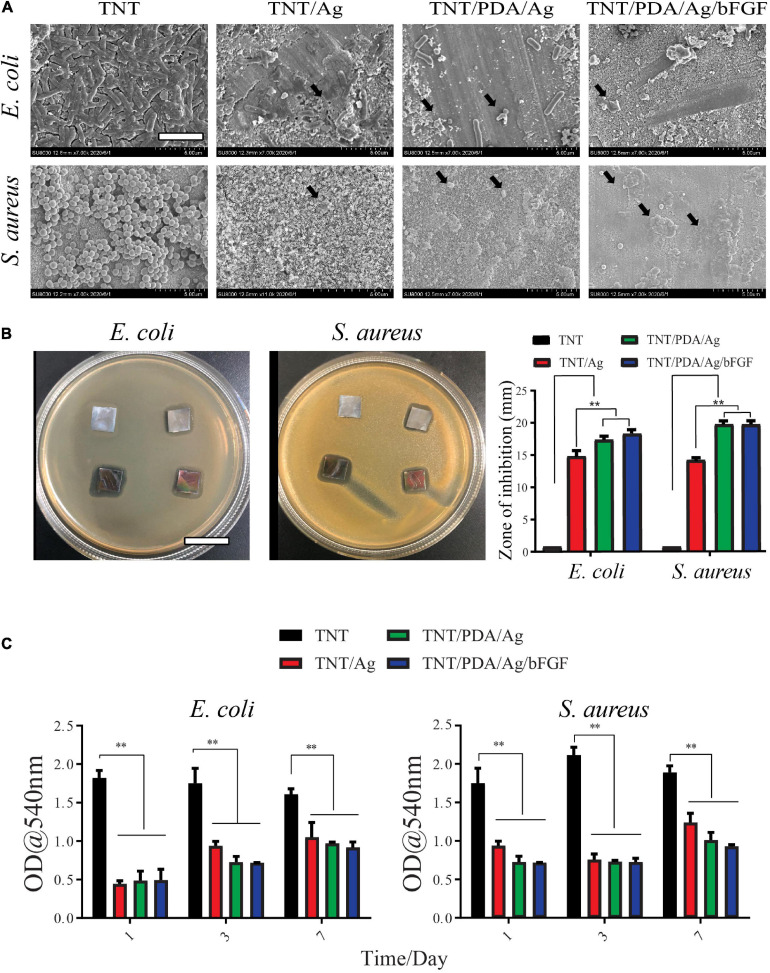
Antibacterial properties of modified Ti samples. **(A)** *Escherichia coli* and *Staphylococcus aureus* were cultured on modified Ti surfaces for 12 h and imaged by SEM. Bacteria with damaged membrane and deformed shape were observed on TNT/Ag, TNT/PDA/Ag, and TNT/PDA/Ag/bFGF (black arrows). Scale bar represents 2 μm. **(B)** Inhibition zone of Ti samples. The bacterial suspension was inoculated, and modified Ti samples were placed on agar plates for 24-h incubation. Bacterial free zone was measured and compared. Both TNT/PDA/Ag and TNT/PDA/Ag/bFGF showed significantly larger inhibition zones. Scale bar represents 2 cm. **(C)** Quantitative analysis of antibacterial ability against *E. coli* and *S. aureus* of Ti samples after being pre-released in sterile phosphate-buffered saline (PBS) for 1, 3, and 7 days. These pre-released Ti samples were placed with bacteria for 24 h. Data are presented as mean ± standard deviation; **p < 0.01.

Gram-negative bacteria, *E. coli*, and gram-positive bacteria, *S. aureus*, were used to examine the antibacterial properties of modified Ti samples. According to SEM observation (Figure 7A), after 12-h incubation on these Ti surfaces, a uniform layer of bacteria, biofilm, formed on TNT surfaces. A rod shape of *E. coli* and a circle shape of *S. aureus* were seen on TNT surfaces. On surfaces of TNT/Ag, TNT/PDA/Ag, and TNT/PDA/Ag/bFGF, the number of bacteria was reduced. Damaged membrane and deformed shape of bacteria were observed.

Modified Ti samples were placed on agar plates for 24 h, where bacteria were inoculated. TNT/Ag, TNT/PDA/Ag, and TNT/PDA/Ag/bFGF have effectively inhibited the growth of *E. coli*, with TNT/PDA/Ag/bFGF being the most powerful. TNT/Ag, TNT/PDA/Ag, and TNT/PDA/Ag/bFGF were able to inhibit the growth of *S. aureus* (Figure 7B).

Ti samples were pre-released in sterile PBS for 1, 3, and 7 days to mimic an application situation, where bacteria arrive after the placement of the implant. These pre-released Ti samples were placed with bacteria for 24 h. Then the inhibition capacity of these pre-released Ti samples on bacterial metabolic activity was assessed by an MTT assay. We found that even after 7-day pre-release, TNT/Ag, TNT/PDP/Ag, and TNT/PDP/Ag/bFGF were still able to suppress the metabolic activity significantly as compared with TNT (Figure 7C). Silver ions (Ag^+^) and their combination have been used in different dental applications such as cement dental resin composites, coatings surfaces, and bone cement (Ai et al., 2017). Ag^+^ and silver nanoparticles are strong bactericidal at low concentrations, presenting an “oligodynamic” impact during the subsistence of toxic ions (Rai et al., 2012). In our work, we adopted silver for its antibacterial and anti-inflammatory features (Feng et al., 2019; Kumar et al., 2019).

### Effect of Titanium Modification on *Porphyromonas gingivalis* Associated With Peri-Implantitis

**FIGURE 8:**
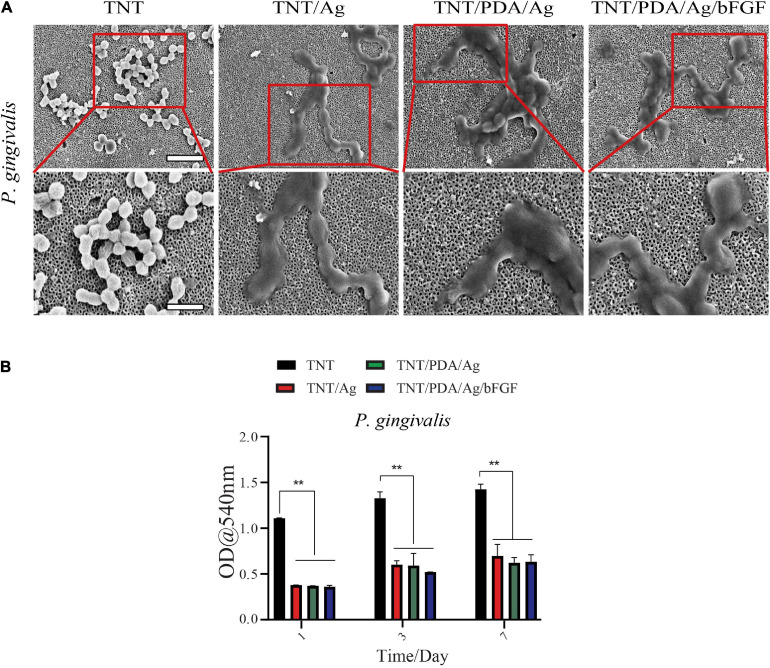
*Porphyromonas gingivalis* associated with peri-implantitis. **(A)** SEM images of titanium-modified surfaces after immersion in *P. gingivalis* culture (scale bar, 5 and 2 μm). After 24 h of incubation with *P. gingivalis*, the TNT/Ag, TNT/PDA/Ag, and TNT/PDA/Ag/bFGF were occupied by clusters of *P. gingivalis*, which formed a visible biofilm. **(B)** Quantitative analysis of antibacterial ability against *P. gingivalis* of Ti for 1 and 3 days. Data are presented as mean ± standard deviation; **p < 0.01.

Scanning electron microscopy images of different titanium modifications are shown in Figure 8A. After 24 h of incubation with *Porphyromonas gingivalis*, the TNT/Ag, TNT/PDA/Ag, and TNT/PDA/Ag/bFGF were occupied by clusters of *P. gingivalis*, which formed a visible biofilm. Ti samples were pre-released in PBS for 1, 3, and 7 days to mimic an application situation. These pre-released Ti samples were placed with *P. gingivalis* for 24 h. Then the inhibition ability of these pre-released Ti samples on *P. gingivalis* metabolic activity was evaluated by an MTT. We found that even after 7-day pre-release, TNT/Ag, TNT/PDP/Ag, and TNT/PDP/Ag/bFGF were still able to suppress the metabolic activity significantly as compared with TNT (Figure 8B). Failures of dental titanium implantation due to a different type of bacterial infection have been increasing (Pye et al., 2009). Furthermore, around 10% of premature failures of titanium have occurred from some bacterial infection a year after implantation, and particularly it has been observed that the main cause for those failures of the implant was bone resorption and site inflammation by different kinds of bacteria such as *P. gingivalis* (van der Reijden et al., 2002). The antimicrobial activities of silver nanoparticles are quite known and broadly documented (Rai et al., 2014). Thus, step-by-step cross-linked silver-coated TNT was applied to decrease *P. gingivalis* infections.

### Anti-inflammatory Properties of Modified Ti Samples

**FIGURE 9:**
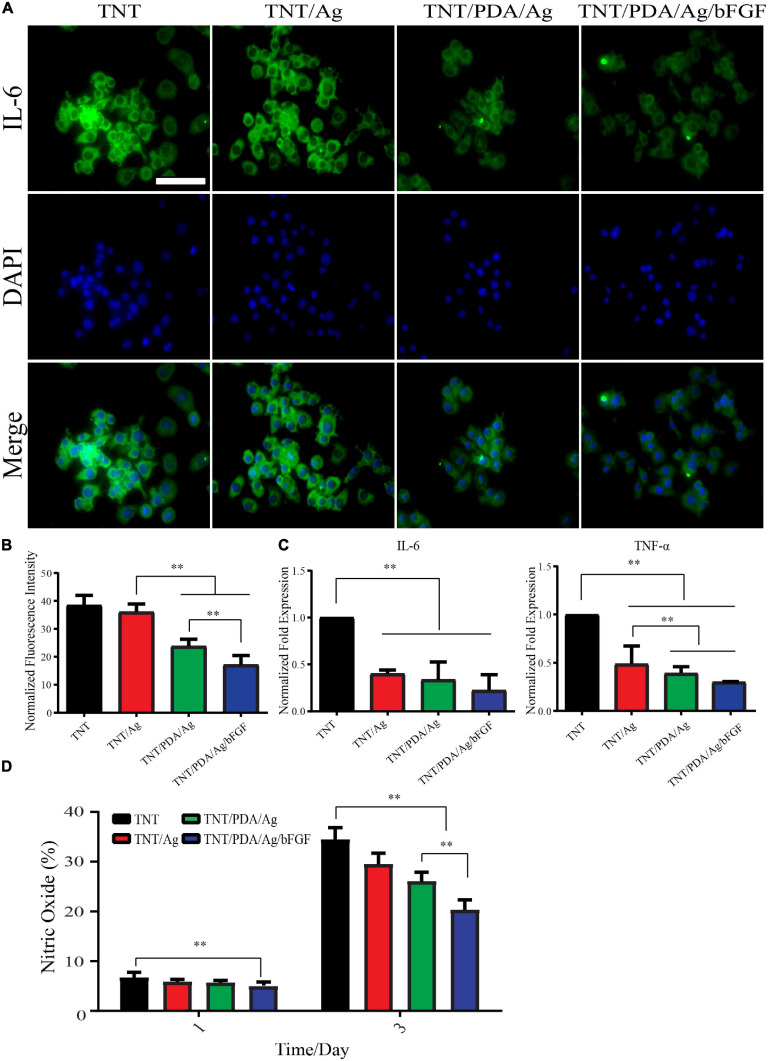
Anti-inflammatory property of modified Ti samples on RAW 264.7 stimulated by lipopolysaccharide (LPS) (500 ng/ml). **(A,B)** LPS exposure of 12 h resulted in elevated expression of IL-6 in macrophages significantly. The expression of IL-6 decreased in the order of TNT, TNT/Ag, TNT/PDA/Ag, and TNT/PDA/Ag/bFGF. Scale bar represents 20 μm. **(C)** After 12 h LPS exposure of RAW 264.7, a significant reduction in gene expression of IL-6 and TNF-α was observed on TNT/Ag, TNT/PDA/Ag, and TNT/PDA/Ag/bFGF. **(D)** To quantify the level of nitric oxide, RAW 264.7 was seeded on sample surfaces with LPS for 1 and 3 days. It was found that TNT/PDA/Ag/bFGF had a good anti-inflammatory effect and decreased nitric oxide production on RAW 264.7. Data are presented as mean ± standard deviation; ^∗∗^*p* < 0.01.

Macrophage RAW 264.7 was used to assess the anti-inflammatory effect of Ti samples. LPS-supplemented medium was applied to cell-adhered Ti surfaces for 12 h. Pro-inflammatory factor, IL-6, was visualized by fluorescence staining (Figure 9A). There was a decreasing trend of the expression of IL-6 in the order of TNT, TNT/Ag, TNT/PDA/Ag, and TNT/PDA/Ag/bFGF. Gene expression of IL-6 and TNF-α also confirmed a significant reduction on cells cultured on TNT/Ag, TNT/PDA/Ag, and TNT/PDA/Ag/bFGF. Both fluorescent and gene results pointed out that TNT/PDA/Ag/bFGF could effectively suppress the inflammatory reaction of macrophages upon an LPS challenge. Ti-modified samples evaluated NO accumulation inhibition in the LPS-activated RAW 264.7 cell. The nitrite production in the RAW 264.7 increased with the LPS at 1 and 3 days. Then NO production was read as nitrite concentration in the medium (50 μl). When compared with TNT/PDA/Ag/bFGF, LPS-induced RAW 264.7 cell released a lower production level of NO in the medium read. It is well-known that the secretion and expression of angiogenic factors such as bFGF are used to inhibit pro-inflammatory factor release. Also, bFGF has increased secretion at sites of acute and chronic inflammation (Shamloo et al., 2018; Albashari et al., 2020). Moreover, the level of bFGF is increasing in the serum and injury tissue of patients with diseases such as asthma, inflammatory bowel disease, and rheumatoid arthritis (Zittermann and Issekutz, 2006). In this article, the anti-inflammatory effects of samples are shown. From Figure 9, it was shown that the expression of pro-inflammatory protein IL-6 gradually increased. However, TNT/PDA/Ag/bFGF showed the opposite in the inflammation regulation (Figures 6, 9A). Also, real-time PCR showed that TNT/Ag and TNT/PDA/Ag mainly decreased the TNF-α and IL-6 expression genes. It was found that TNT/PDA/Ag/bFGF had good anti-inflammatory activity and decreased nitric oxide production on RAW 264.7 (Figures 9B,C). bFGF can be a great positive regulator in acute and chronic inflammation. Furthermore, the IL-6 and TNF-α inhibition represent a positive part in the osteogenesis and without inducing prolonged inflammation (Bastidas-Coral et al., 2016). In *in vitro* works, it was found that Ag ions and nanoparticles decrease secretion of TNF-α from macrophage cells to suppress inflammation (Lappas, 2015). Another paper has shown that AgNPs on titanium surfaces mainly regulated the macrophage polarization (Pratsinis et al., 2013).

## Conclusion

PDA/Ag/bFGF coating was successfully applied on the titanium surface. It was demonstrated that the surface modifications on Ti, including nanotube formation with coatings, could promote the slow release of bioactive bFGF. Our study also showed that cross-linking silver and bFGF on modified Ti surface at nanoscale had strong early anti-infiammatory and antibacterial properties. DPSCs cultured on this modified Ti (TNT/PDA/Ag/bFGF) could achieve an enhanced osteogenic differentiation. Together, our findings provide an alternative for the multifunctional titanium implants and expand the application of cross-linking of antibacterial with growth factor in bioengineering fields.

## Data Availability Statement

The original contributions presented in the study are included in the article/supplementary material, further inquiries can be directed to the corresponding author/s.

## Ethics Statement

The studies involving human participants were reviewed and approved by the School and Hospital of Stomatology, Wenzhou Medical University (No. WYKQ2018008). The patients/participants provided their written informed consent to participate in this study.

## Author Contributions

QY and AA: design. AA, MA, YX, JA, and FH: methodology and validation. YZ, KZ, and LL: data curation and analysis. AA and YH: writing—original draft preparation. QY andJW: writing—review and editing. LL, YH, and QY: funding acquisition. All authors have read and agreed to the published version of the manuscript.

## Conflict of Interest

The authors declare that the research was conducted in the absence of any commercial or financial relationships that could be construed as a potential conflict of interest.
